# Reproductive health service use and associated factors among youths in Becho district, southwest Ethiopia

**DOI:** 10.3389/fpubh.2023.1062325

**Published:** 2023-03-02

**Authors:** Teshome Tolosa Waga, Muluneh Shuremu, Asrat Zewdie, Abeza Mitiku Kera, Gutama Haile Degefa

**Affiliations:** ^1^Ilu Aba Bor Zone Health Department, Mattu, Oromia Region, Ethiopia; ^2^Department of Public Health, Mattu University, Mattu, Ethiopia; ^3^Department of Environmental Health and Technology, Jimma University, Jimma, Ethiopia

**Keywords:** Ethiopia, factors, Ilu Aba Bor, reproductive health service, utilization, youth

## Abstract

**Background:**

Young people are less informed, less experienced, and less at ease when it comes to accessing reproductive health services than adults. Though youth-friendly services are designed to accommodate the unique needs of youth, in developing countries like Ethiopia, studies on the level of utilization of reproductive health services are limited.

**Objectives:**

This study determined the level of reproductive health (RH) service use and associated factors among youths.

**Methods:**

A community-based cross-sectional study was conducted in Becho district, Illubabor zone, southwest Ethiopia. A multistage random sampling technique was used to select 702 youths, regardless of their marital status. A pre-tested interviewer-administered questionnaire was used to collect data. The questionnaire includes questions on socio-demographic characteristics, sexual and reproductive health characteristics, knowledge, and components of RH assessment. The data was entered into Epidata version 3.1 and analyzed using SPSS version 22. Multivariable binary logistic regression analysis was used to identify factors associated with the utilization of reproductive health services at a *p* < 0.05.

**Results:**

A total of 647 youths participated in the study, constituting a response rate of 92.1%. Male youths made up 51.5% of the respondents, with an average (±SD) age of 19.38 (±2.69) years. Reproductive health (RH) services were utilized by 43.9% of youths. Knowledge of RH services (AOR = 4.11; 95% CI: 2.77, 6.09), discussion with family (AOR = 2.18; 95% CI: 1.38, 3.45), history of sexual exposure (AOR = 2.94; 95% CI: 1.95, 4.43), shorter distance from a health facility (AOR = 2.42; 95% CI: 1.63, 3.57), and history of reproductive health problems (AOR = 2.4; 95% CI: 1.34, 4.31) were associated with RH service utilization.

**Conclusion:**

The use of reproductive health services among youths is found to be low. Knowledge about reproductive health services, discussion with parents, sexual exposure, distance, and previous experience with reproductive health problems shaped the utilization of RH services by youth. Improving knowledge through information dissemination, creating awareness to increase parent-child intimacy, and expanding health services should be emphasized.

## Introduction

The use of reproductive health services is an important component in preventing various sexual and reproductive health problems in adolescents (1, 2). Youths are individuals aged between 15 and 24 who are at a life stage that is undergoing significant physiological, psychological, and social changes (3). According to the United Nations, youths account for 15.5% of the global population (4), and in Sub-Saharan Africa, 19.9% of the total population were youths in 2020 (4). In Ethiopia, youths represent a large segment (21.3%) of the population (5).

Countries have been encouraged to implement youth-friendly service programs since the 1994 International Conference on Population Development (ICPD), which must be effective, accessible, acceptable, safe, and affordable, as well as protect the youth's privacy, confidentiality, respect, and informed choice (6–8).

In pursuing the youth reproductive health agenda and improving the health status of this population group, the government of Ethiopia has developed a comprehensive national adolescent and youth reproductive health strategy (NAYRHS) with a focus on implementing youth-friendly services for the achievement of the second Growth and Transformation Plan (GTP) and Sustainable Development Goals (SDG) (3, 9).

The essential package of reproductive health services offered for youth in Ethiopia includes family planning services, diagnosis and management of sexually transmitted infections, testing services for pregnancy and HIV/AIDS, abortion and post-abortion care services, maternal health services (antenatal care, delivery service, postnatal care, and PMTCT), information and counseling on sexual and reproductive health issues, including sexuality [information, education, and communication (IEC)], and condom promotion and provision (2, 3).

The pooled prevalence of youth-friendly reproductive health service utilization in Ethiopia was 42.73% (95% CI: 35.38–50.09) (10). Youths are at great risk of reproductive health problems due to their overall situation of poor socioeconomic status, unfriendliness of available services, poor knowledge of the environment, and harmful traditional practices (2, 3, 11). The major physical, cognitive, sexual, and social changes that occur during adolescence affect youth's sexual behaviors, which in turn compromise their health-seeking behavior (3, 12). They are less informed, have less experience, and are less comfortable accessing reproductive health services than adults (6, 13–15).

The WHO has estimated that 70% of premature deaths among adults are largely due to behaviors initiated during adolescence (1). Globally, about 42% of all reproductive health problems occur among people aged 15–24 years, with the majority (80%) living in sub-Saharan Africa (8).

In Ethiopia, where about 64% of young people start sexual intercourse before the age of 18 (16), combined with the inadequate uptake of family planning services, youths are exposed to the top sexual and reproductive health problems like unwanted pregnancy, sexually transmitted infections, including HIV/AIDS, and unsafe abortion, which ultimately results in child and maternal mortality and socioeconomic repercussions (15, 17, 18).

Various factors affecting youth's access to reproductive healthcare continue to exist today. These factors include inconvenient service locations, limited operating hours, provider attitudes, a lack of service quality, confidentiality and privacy, and high service costs (19–22). Most youths are unaware of the types of sexual and reproductive health services that are available in existing service outlets, who provides the services, and how they would be served (23–26).

Despite Ethiopia's efforts to implement a national adolescent and youth reproductive health strategy (3) and a standard youth reproductive health service (2) with a specific focus on improving the use of research and evidence for decision-making for better RH care and access to and utilization of RH services, which has become a primary concern of sexual and reproductive health rights in the country (2, 25), the existing health services are insufficient and lack evidence (3, 6, 13).

Regardless of the fact that youths constitute one-third of the population and experience significant negative health-related outcomes in Ethiopia, studies on the extent of reproductive health service use based on standards of comprehensive youth reproductive health service packages and the related factors are limited. Even though there were other studies, including a systematic review, in the country, the comprehensive RH service packages for youth that are outlined in the standard for RH service delivery in the country (2) were not addressed. Particularly, the existing systematic review study in the country only considered and directly used the components used in a single cross-sectional study conducted on youth in school in Ethiopia (27), which was not based on standards to measure RH service utilization.

The current study used the comprehensive RH service packages for young people listed in the national standard guidelines prepared to guide and prescribe the minimum service delivery package on youth-friendly reproductive health services in the country. Thus, this study was designed to determine the level of utilization of reproductive health services based on the national standard for youth RH service utilization and identify socio-demographic, sexual and reproductive health, and health service accessibility factors associated with the utilization of reproductive health services among youths in Becho district, southwest Ethiopia. The findings of this study can be used by local health authorities and youth health planners to design appropriate youth-targeted interventions in the study area. Besides, it could be used as an input for evaluating the implementation of the national adolescent and youth reproductive health strategy in the study area.

## Methods and materials

### Study design and setting

A community-based cross-sectional study was conducted in the Becho district, Iluababor zone, southwest Ethiopia from June 3 to July 30, 2020. The district is one of the 14 districts found in the Ilubabor zone in Oromia Regional State, located in the southwestern part of Ethiopia. It is located 621 km from Addis Ababa and 21 km from Mattu, the capital of the Illubabor zone. There are 16 rural kebeles (the lowest government administrative unit next to district in Ethiopia) and one urban kebele. The district has a population of 52,388 as calculated based on the 2007 census (28). The total number of youths in the district during the study period was 8,241. From these, the number of females was 3,978 (47.9%). The total number of married youths was 2,955 (35.8%), of whom 1,891 (67%) were married women. There are two public health centers in the district that are in charge of providing reproductive health services to youth (29).

### Study population

The study population included youths aged 15–24 years, regardless of marital status, living in six randomly selected kebeles, five rural and one urban kebele in the Becho district. Youths who were temporary residents (those who lived for <6 months) in the district were excluded.

### Sample size and sampling techniques

The sample size was calculated using a single population proportion formula with the following assumptions: From an estimate of the proportion (p) of youth-friendly service utilization from a study conducted in the Hadiya zone, 70.6% (15) was used as the basis for the calculation, n = minimum sample size, 5% margin of error tolerated (d), and 95% confidence interval (z) was used as the basis for the calculation $n=\left(z\;\frac{\alpha }{2\;}{)}^{2}\right)p\left(\frac{1-p}{d^{2}}\right)=1.96^{2}(\frac{0.294^{*}0.706)}{0.05^{2}}$ = 319. Using a design effect of two and considering a 10% non-response rate, the sample size for the level of youth reproductive health service utilization was found to be 702. Sample size was also calculated for factors associated with reproductive health service utilization based finding s of previous studies. Accordingly, information about reproductive health services, sources of information, and sexual exposure were important factors (13, 14) affecting reproductive health service utilization among youths and used in the sample size calculation which yield a sample size of 666. The largest sample size from the two, which was 702, was considered as the final sample size.

**Figure 1:**
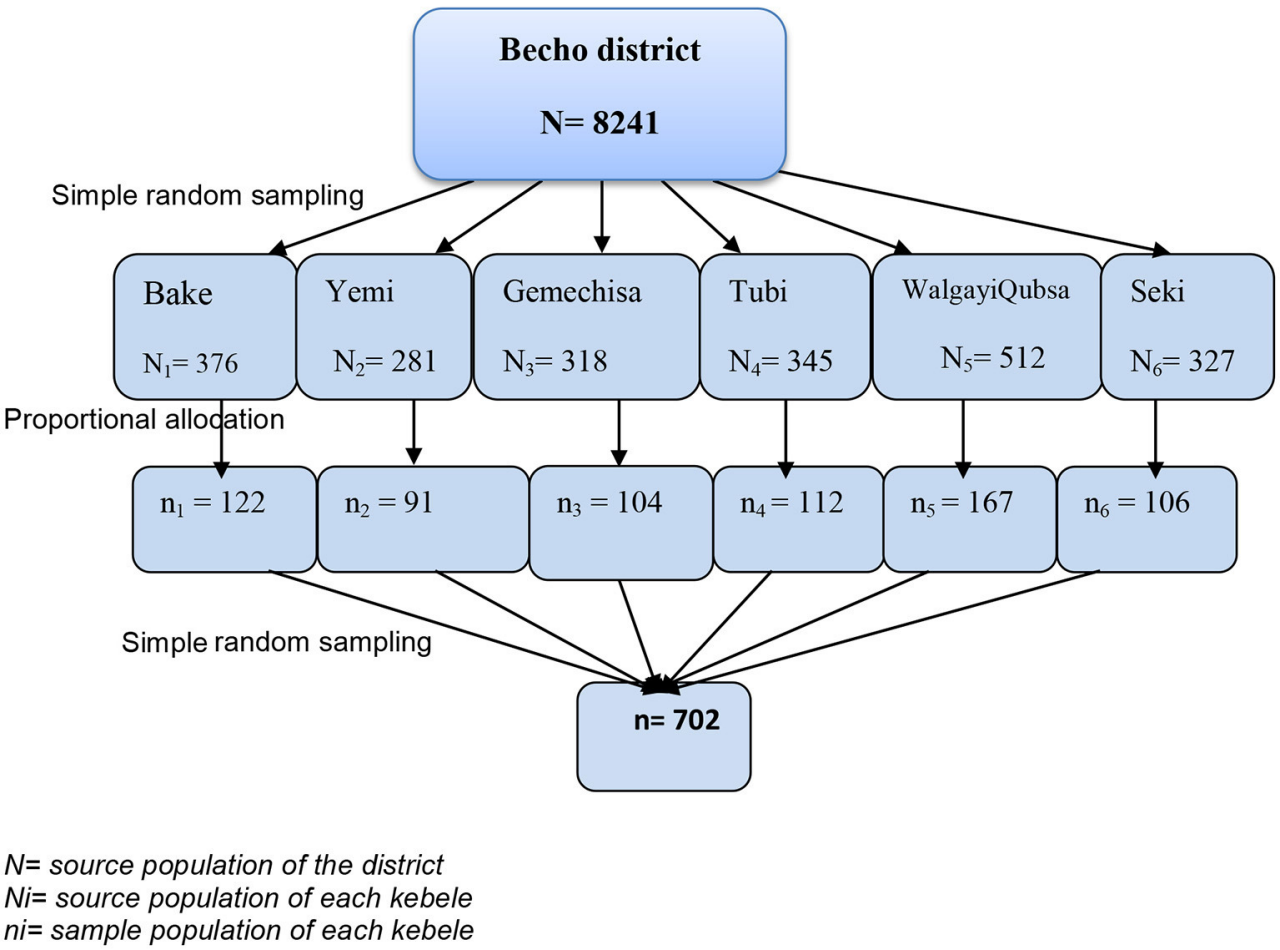
Schematic presentation of the sampling procedure.

A multistage random sampling technique involving two stages was used to select the study participants from all youths living in the Becho district. In the first phase, a simple random sampling technique was used to select six kebeles from 17 kebeles in the district. Secondly, households containing young people were identified from the register of households registered for onchocerciasis prophylaxis by the Multi-Drug Administration that was used to prepare the sampling frame. Youths were selected by using a simple random sampling technique (6, 30, 31) from the register. The total sample size (*n* = 702) was proportionally allocated to the youth population size of each randomly selected kebele, using the proportionate sampling method (Figure 1).

### Data collection tools and procedures

An interviewer-administered a structured questionnaire, which was adopted from different kinds of literature (14, 15, 24, 32), and was used to collect quantitative data on youths' socio-demographic characteristics, personal characteristics related to sexual and reproductive health, knowledge about reproductive health services, and level of reproductive health service utilization, through home-to-home visits. The questionnaire was developed in English and translated into Afan Oromo by a language expert and back-translated to English to check for its consistency. Ten trained clinical nurses were recruited for data collection, and two B.Sc. nurses supervised the data collection process.

### Measurement

Utilization of reproductive health services was the outcome variable. It was measured by asking participants about using one of the seven basic components of youth reproductive health services based on the national Standards on Youth Friendly Reproductive health Services (2) such as family planning information service including emergency contraceptive methods, diagnosis and management of sexually transmitted infections, testing services for pregnancy and HIV/AIDS, abortion and post abortion care service, maternal health service (antenatal care, delivery service, postnatal care and PMTCT), information and counseling on sexual and reproductive health issues including sexuality [information, education, and communication (IEC)] and condom promotion and provision in the last 12 months preceding the survey (6, 11, 33). Utilization of RH services was considered “yes (1)” if any of these seven services were utilized or “no (0)” otherwise. The explanatory variables included socio-demographic characteristics (age, gender, marital status, educational status, family education), knowledge about reproductive health services, source of information, history of sexual exposure, discuss about reproductive health issues with family, history reproductive health problem, distance from the healthcare facility, convenient location of the health facility, and payment for youth-friendly services). Knowledge of reproductive health services was assessed using yes (1) or no (0) responses to eight-item questions about reproductive health services (14, 24, 34). The median knowledge score was taken as the cut-off point for deciding the level of knowledge based on the sum of the scores. Values above the median were coded as “1” for good knowledge, while values below the median were coded as “0” for poor knowledge. Sexual exposure was measured by asking whether the youth had at least one sexual contact in their life until the time of data collection (16). In this study, “long distance” was defined as traveling more than 60 min to reach health facilities, whereas convenient location” was defined as the location of a reproductive health service providing health facility that cannot easily expose the youth to the adult population (6).

### Data management and analysis

The collected data was edited, coded, and entered into a computer using Epi-Data version 3.1 and exported to SPSS version 22 for analysis. A descriptive analysis was conducted to check for outliers and consistency, and to identify missed values for independent variables. Simple binary logistic regression analysis was employed to see the association between each explanatory variable and the outcome variable. Variables were initially evaluated for the effect of confounding by using pseudo-regression analysis, which can handle large number of confounders simultaneously. Then, variables with a change in the odds ratio (OR) of 10% or more upon including a confounder in the model were controlled for by leaving it in the model, and if a 10% change in OR is not observed, variables were removed from the model, as they do not need to be controlled for (35, 36). Variables with a valid confidence interval and *p* < 0.25 in the bivariate analysis were entered as the independent variables in the multivariable binary logistic regression model, with a *p*-value of 0.05 considered statistically significant for all independent variables in the final model. Fitness of the final model was checked by the indication of a *p*-value of 0.313 in the Hosmer and Lemeshow goodness of fit test.

## Results

### Socio-demographic characteristics of the participants

From a total of 702 youths, 647 youths participated, with a response rate of 92%. More than half (51.5%) of respondents were male. The mean (±SD) age of the respondents was 19.38 (±2.69) years, with 55.3% of them in the age range between 15 and 19 years old. About three in 5 (64%) youths were single, and 44% were students. Nearly half (48.4%) of the study participants reported living with their father and mother (refer to Table 1).

**Table 1:** Socio-demographic characteristics of youths in Becho district, southwest Ethiopia, 2020.

	**Category**	**Frequency (*n* = 647)**	**Percent**
Age category	15–19 years	358	55.3
	20–24 years	289	44.7
Gender	Male	333	51.5
	Female	314	48.5
Marital status	Never married	415	64.1
	Ever married	232	35.9
Educational status	Cannot read and write	32	5.7
	Can read and write	57	8.0
	Primary	267	41.3
	Secondary	240	37.1
	College and above	51	7.9
Occupation/activity status	Farmer	139	21.5
	Merchant	73	11.3
	Government employee	28	4.3
	Student	287	44.4
	Daily laborer	104	16.1
	Housewife	16	2.5
Living arrangement	Married	232	35.8
	Live with boy/girlfriend	102	15.8
	With parents	313	48.4
Father education	Cannot read and write	145	22.4
	Can read and write	154	23.8
	Primary	200	30.9
	Secondary	87	13.4
	College and above	61	9.4
Mother education	Cannot read and write	284	43.9
	Can read and write	138	21.3
	Primary	168	26.0
	Secondary	34	5.3
	College and above	23	3.6

### Characteristics regarding sexual and reproductive health and health service accessibility

Two-thirds (66.8%) of study participants had awareness of reproductive health services, and their primary sources of information were health professionals (31.4%), followed by friends (27.4%). More than three in 5 (62%) of them had a sexual partner, and the majority of them (79.6%) had only one sexual partner. About 62% of youths reported having ever had sex. More than one in ten of the participants (11.3%) had a history of reproductive health problems; with the majority (67.1%) being sexually transmitted infections. The main nearby youth reproductive health services providing health facilities reported by the participants were health centers (55.6%), followed by health posts (33.6%). More than half of them (58.1%) reported that the location of the health facilities was convenient. Of the study participants, the majority (80%) of them had not discussed reproductive health issues with their families, as indicated in Table 2.

**Table 2:** Characteristics regarding sexual and reproductive health and health service accessibility among youths in Becho district, southwest Ethiopia, 2020.

	**Category**	**Frequency (*n* = 647)**	**Percent**
Awareness about RH service^*^	Yes	432	66.8
	No	215	33.2
Major source of information	Parents	64	9.9
	School	93	14.4
	Heath providers	203	31.4
	Friends	177	27.4
	Media	110	16.9
Ever had a sexual partner	Yes	402	62.1
	No	245	37.9
Number of sexual partners (*n* = 402)	One	319	79.4
	More than one	83	20.6
Ever had sexual intercourse	Yes	399	61.7
	No	248	38.3
History of RH problem	Yes	73	11.3
	No	574	88.7
Type of RH problem faced (*n* = 73)	STI	49	67.1
	Unwanted pregnancy	24	32.9
Discussion about RH with family	Yes	128	19.8
	No	519	80.2
Payment asked for RH service (*n* = 284)	Yes	135	47.5
	No	149	52.5
Convenient RH service location	Yes	376	58.1
	No	271	41.9
Nearby RH service delivery point	Health post	217	33.6
	Health center	360	55.6
	Private clinic	70	10.8
Distance from health facility	Less than 60 minutes	472	73.4
	More than 60 minutes	175	26.6

* RH, reproductive health.

### Knowledge about reproductive health services among youths

More than a quarter (27%) of the youths didn't know that sexual health education and prevention information for young people are provided where confidentiality and privacy are assured. A majority (84.7%) of youths know that reproductive health service is a right. Only about quarters (25.2%) of youths know that sexuality counseling is given for the client's sexual health concerns or needs and desired sexuality, reproductive, or contraceptive preferences. Generally, 378 (58.4%) youths had good knowledge about reproductive health services, as indicated in Table 3.

**Table 3:** Knowledge about reproductive health services among youths in Becho district, southwest Ethiopia, 2020.

	**Category**	**Frequency (*n* = 647)**	**Percent**
Confidentiality and privacy of sexual health education	Yes	472	73
	No	175	27
Post-abortion care and provision of contraceptive	Yes	250	38.6
	No	397	61.4
Diagnosis, treatment, and follow-up for STIs^*^	Yes	160	24.7
	No	487	75.3
Reproductive health right	Yes	548	84.7
	No	99	15.3
Voluntary counseling and testing for HIV/AIDS	Yes	320	49.5
	No	327	50.5
Safe abortion care	Yes	91	14.1
	No	556	85.9
Antenatal, intra-natal, and post-natal care	Yes	369	57.1
	No	278	42.9
Sexuality counseling	Yes	163	25.2
	No	484	74.8
Knowledge about RH services^*^	Good	378	58.4
	Poor	269	41.6

* Composite variable.

### Utilization of reproductive health services among youths

**Figure 2:**
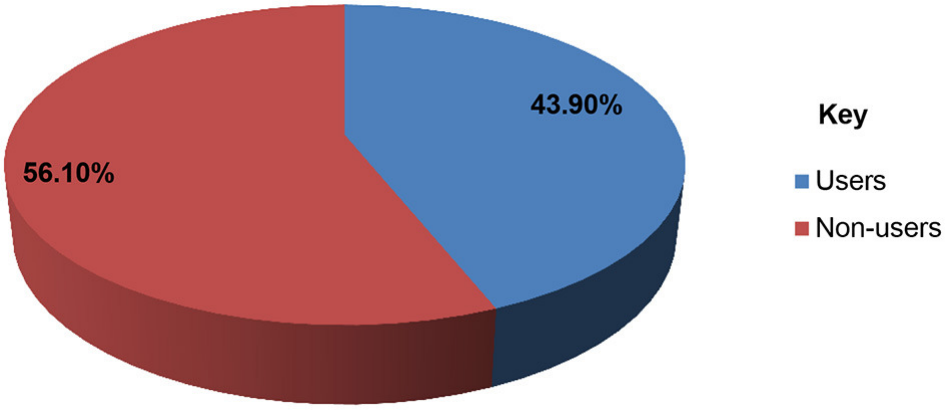
The level of reproductive health services utilization among youths in Becho district, southwest Ethiopia, 2020.

Though 58.4% of the youths had good knowledge about reproductive health services, only 43.9% of them utilized at least one of the reproductive health services in the last 12 months preceding the survey (Figure 2). The major services utilized by respondents were contraceptive use (18.5%), HIV testing and counseling (18.4%), followed by condom use (9.4%) (Table 4).

**Table 4:** Reproductive health service utilization among youths in Becho district, southwest Ethiopia, 2020.

	**Category**	**Frequency (*n* = 647)**	**Percent**
Contraceptive use	Yes	120	18.5
	No	527	81.5
Diagnosis and treatment for STIs	Yes	58	8.9
	No	589	91.1
HIV testing and counseling service	Yes	119	18.4
	No	528	81.6
Abortion services (*N* = 314)	Yes	13	4.1
	No	301	95.9
Maternal health service (ANC, delivery, PNC) (*N* = 314)	Yes	21	6.7
	No	293	93.3
Counseling service	Yes	7	1.1
	No	640	98.9
Condoms distribution	Yes	61	9.4
	No	586	90.6
Utilization of RH service	Users	284	43.9
	Non-users	363	56.1

STI, sexually transmitted infection; RH, reproductive health.

### Factors associated with utilization of reproductive health services

After controlling for potential confounders, having good knowledge of reproductive health services, a history of sexual exposure, discussing reproductive health issues with family, previous experiences with reproductive health problems, and distance from a health facility were significantly associated with youth reproductive health service utilization in the final model.

Youth who had good knowledge of reproductive health services were four times more likely to use them than their counterparts (AOR = 4.11; 95% CI: 2.77, 6.09). Youth who discussed reproductive health issues with their families were two times more likely to use reproductive health services than their counterparts (AOR = 2.18; 95% CI: 1.38, 3.45). Youth who had ever had sexual contact, on the other hand, were about three times more likely to use youth reproductive health services as those who had never had sexual contact (AOR = 2.94; 95% CI: 1.95, 4.43). Youths who spent <60 min on the way to a health facility had more than two times the likelihood of utilizing reproductive health services than youths who spent more than 60 min (AOR = 2.42, 95% CI: 1.63, 3.57). In this study, young people who had ever faced any reproductive health problem were more than two times more likely to utilize youth reproductive health services than those who had history of a reproductive health problem (AOR = 2.4, 95% CI: 1.34, 4.31) (refer to Table 5).

**Table 5:** Factors associated with utilization of reproductive health service utilization among youths in Becho district, southwest Ethiopia, 2020.

	**RH service use COR 95% CI AOR 95% CI**			
	**User** ***n*** **(%)**	**Non-user** ***n*** **(%)**		
**Age category**				
15–19 years	135 (37.7)	223 (62.3)	1.00	1.00
20–24 years	149 (51)	140 (49)	0.56 (0.41, 0.78)	0.78 (0.52, 1.15)
**Gender**				
Male	132 (39.6)	201 (60.4)	1.00	1.00
Female	152 (48.4)	162 (51.6)	0.7 (0.51, 0.96)	0.6 (0.41, 1.086)
**Marital status**				
Ever married	132 (56.9)	100 (43.1)	0.43 (0.31, 0.60)	0.80 (0.519, 1.243)
Never married	152 (36.6)	263 (63.4)	1.00	1.00
**Knowledge about RH services**				
Good	208 (55.0)	170 (45.0)	3.1 (2.22, 4.33)	4.11 (2.77, 6.09)^*^
Poor	76 (28.3)	193 (71.7)	1.00	1.00
**Discuss RH issues with family**				
Yes	76 (59.4)	52 (40.6)	2.18 (1.47, 3.24)	2.18 (1.38, 3.45)^*^
No	208 (40.1)	311 (59.9)	1.00	1.00
**Distance from health facilities**				
< 60 min (short)	181 (49.3)	186 (50.7)	1.67 (1.2, 2.29)	2.42 (1.63, 3.57)^*^
>60 min (long)	103 (36.8)	177 (63.2)	1.00	1.00
**History of sexual exposure**				
Yes	222 (55.6)	177 (44.4)	3.76 (2.65, 5.33)	2.94 (1.95, 4.43)^*^
No	62 (25)	186 (75)	1.00	1.00
**History of RH problem**				
Yes	50 (68.5)	23 (31.5)	3.16 (1.87, 5.32)	2.4 (1.34, 4.314)^*^
No	234 (40.8)	340 (59.2)	1.00	1.00

* Significant at p < 0.05. CI, confidence interval; COR, crude odds ratio; AOR, adjusted odds ratio; RH, reproductive health.

## Discussion

The purpose of this study was to assess the level of reproductive health service utilization and associated factors among youths in Becho district, Illubabor zone, southwest Ethiopia. Accordingly, about 44% of youths utilized at least once of the reproductive health services in the last 1 year preceding the survey. Several factors were identified to affect the reproductive health service use among youths.

The rate in this study is comparable to the Ethiopian national rate (42.73%) (10) based on the systematic review, but is higher and lower compared to studies conducted in different parts of Ethiopia. Studies conducted in the following districts found lower rates: Hadiya, 38.5% (15), Mecha district, 18.4% (33), and Bahir Dar, 32% (11). This difference may be due to a difference in accessibility of health services, knowledge of youths about reproductive health services, and history of reproductive health problems, as well as progress in the promotion and expansion of reproductive health services for young people in recent years, which might contribute to the higher utilization.

In contrast, this study's finding of the level of RH service utilization is lower than the results of studies conducted in the following parts of Ethiopia: Harar (64%) (6), Bale (46.9%) (25), Kachabira (47.2%) (37), Mekele (69.1%) (38), and North Shewa of the Amhara region (79.9%) (39). The difference might be attributed to the youth's lack of knowledge about reproductive health services, the absence of parental monitoring, parental involvement, or communication with their youth, as well as the long distance from health facilities that would make it difficult to utilize reproductive health services in the study area.

This study's finding of the level of RH service utilization is also lower than the studies done in other African countries, such as Nigeria (51%) (34) and Ghana (55.8%) (40). This difference may be attributable to the difference in the study area, socio-cultural factors, service delivery systems, and the openness of the study participants between areas. This could also be explained by the fact that Ethiopian society considers youths to be too young to visit a health institution due to cultural influences, and visiting the institution for specific sexual and reproductive health services may be considered shameful.

In this study, knowledge of youth reproductive health services was linked to youth reproductive health service utilization. This result is in line with the studies conducted in Ethiopia (14, 15, 37), Nigeria (34), and Ghana (40). The possible explanation for this is that young people who are knowledgeable about the availability and use of reproductive health services will freely decide to use the reproductive health services they prefer. Besides, youth who are knowledgeable about RH services are so because they had a RH problem, which made them seek out the services.

In this study, parental involvement or communication with their youth influenced the utilization of reproductive health services. This is in line with a study conducted in Gondar, Ethiopia (14) and North Shewa, Ethiopia (39). This can be justified by the fact that parent-child relationships may impact a young person's decision to use youth reproductive health services. Communication between parents and children is a means of transmitting sexual values, beliefs, and knowledge from parents to their children. When youths feel connected to their families, they are more likely to engage in healthy behavior, which may help them deal with reproductive-related issues. Greater openness from parents on the topic of reproductive health (less taboo and stigmatization) may also lead to more communication and, thus, greater knowledge and use of RH services.

Youths who live close to a health facility are more likely to use reproductive healthcare services than those who live a long distance away. Supporting this finding, a study conducted in Nigeria (41) revealed that the further a patient lives from a health facility, the less likely they are to utilize the services. A study in Kenya also identified a long distance from the facility or care source as a barrier to the use of health services (42). This is because the preferred care source is often the closest one. Moreover, in the African context, the principal barriers to accessibility are transport and cost, so distance is mostly reported as a single obstacle to the utilization of healthcare services (13, 22).

In this study, sexual exposure was another factor that was associated with youth reproductive health service utilization. This finding is comparable with the studies conducted in Hadiya (15), Bale (25), Mecha district (33), Awabel (43), and North Shewa (39). This can be justified due to the difference in risk perception. Respondents who had sexual contact might have a higher perception of the risk of reproductive health problems than their counterparts, and youths who were sexually active might need services to avoid being exposed to risky sexual activities, as they might need information and counseling services on how to avoid risky behavior.

In this study, like in other similar studies (11, 24, 37), utilization of reproductive health services was higher among youths with a reproductive health problem. This can be justified due to the fact that respondents who had sexual contact might have a higher perception of the risk of reproductive health problems than their counterparts, and sexually active young people might need services to avoid being exposed to risky sexual activities just as they might need information and counseling services on how to avoid risky behavior.

This study situated the current level of reproductive health service utilization and identified factors contributing to it, which could be an input for the evaluation of the outcome of the 2016–2020 national adolescent and youth reproductive health strategy in the study area. The government's investment in and ownership of adolescent and youth sexual and reproductive health (AYSRH) programs and services are strategic means to improve the future development of the country (44). As long as advocating for adolescent and youth health as a national priority remains important, we must focus on generating evidence and honing strategies to guide AYSRH service provision. The findings in this study can be used as evidence of the effectiveness of youth-friendly services and interventions that can inform policies and AYSRH programs as we continue to progressively build and scale up what has worked to create access to AYSRH programs and monitor and ensure the quality of youth-friendly services. These findings could serve as a baseline for future research involving qualitative designs, programs, and policy interventions.

### Limitations of the study

The cross-sectional design of this study precluded drawing causal inferences between explanatory factors and reproductive healthcare service use. Since studies on reproductive issues are very sensitive, they might result in social desirability bias, which could underestimate or overestimate the magnitude of reproductive health service utilization. There is the possibility of missing other factors related to the perspectives of health workers or parents since they were not included in the study.

## Conclusion

The use of reproductive health services among youths observed in this study was found to be comparable with the national level. Good knowledge of reproductive health services, discussion with parents or peers, having a history of reproductive health problems, and living in close proximity to health facilities were among the factors associated with the utilization of reproductive health services among youths. It is crucial to offer this group of young people adequate reproductive health information and friendly and accessible reproductive health services.

## Recommendations

Health education to improve knowledge and parent-child intimacy in discussing reproductive health issues and the promotion of health services shall be emphasized in the study area. It is critical to improve the accessibility of reproductive health services to young people through the integration of youth-friendly services into health extension packages or the establishment of youth-friendly service centers at the kebele level. Of note, educating youths about their reproductive health rights and expanding health services is recommended to raise the level of reproductive health service use.

Future studies supported by qualitative methods involving parents, teachers, and service providers should be carried out to better understand the cultural and supply-side barriers to creating conditions for providing friendly and efficient RH services to youth.

## Data availability statement

The original contributions presented in the study are included in the article/supplementary material, further inquiries can be directed to the corresponding author.

## Ethics statement

The study involved human participants, and was reviewed and approved by Ethical Review Board of Mattu University, College of Health Science with approval number RCS/023/2019. Written informed consent to participate in this study was provided by the participants' legal guardian/next of kin.

## Author contributions

TTW made substantial contributions to the conception or design of the work or the acquisition, analysis, or interpretation of data for the work. MS, AZ, AMK, and GHD made substantial contribution to the development of data collection tools, formal analysis, supervision, and drafting of the first work and revising it critically for important intellectual content. All authors are involved in the final approval of the version to be submitted to the journal.
